# A Methodology of Condition Monitoring System Utilizing Supervised and Semi-Supervised Learning in Railway

**DOI:** 10.3390/s23229075

**Published:** 2023-11-09

**Authors:** Jaeseok Shim, Jeongseo Koo, Yongwoon Park

**Affiliations:** 1 Complex Research Center for Materials & Components of Railway, Seoul National University of Science and Technology, Seoul 01811, Republic of Korea; shimjaeseok@seoultech.ac.kr; 2 Department of Railway Safety Engineering, Seoul National University of Science and Technology, Seoul 01811, Republic of Korea; koojs@seoultech.ac.kr; 3 A2Mind, 213, Toegye-ro, Jung-gu, Seoul 04557, Republic of Korea

**Keywords:** condition monitoring, STFT (short time Fourier transform), CNN, supervised learning, LeNet-5, ResNet-20, MobileNet, semi-supervised learning, Deep One Class Classification, TCMS (train control and management system)

## Abstract

In this paper, research was conducted on anomaly detection of wheel flats. In the railway sector, conducting tests with actual railway vehicles is challenging due to safety concerns for passengers and maintenance issues as it is a public industry. Therefore, dynamics software was utilized. Next, STFT (short-time Fourier transform) was performed to create spectrogram images. In the case of railway vehicles, control, monitoring, and communication are performed through TCMS, but complex analysis and data processing are difficult because there are no devices such as GPUs. Furthermore, there are memory limitations. Therefore, in this paper, the relatively lightweight models LeNet-5, ResNet-20, and MobileNet-V3 were selected for deep learning experiments. At this time, the LeNet-5 and MobileNet-V3 models were modified from the basic architecture. Since railway vehicles are given preventive maintenance, it is difficult to obtain fault data. Therefore, semi-supervised learning was also performed. At this time, the Deep One Class Classification paper was referenced. The evaluation results indicated that the modified LeNet-5 and MobileNet-V3 models achieved approximately 97% and 96% accuracy, respectively. At this point, the LeNet-5 model showed a training time of 12 min faster than the MobileNet-V3 model. In addition, the semi-supervised learning results showed a significant outcome of approximately 94% accuracy when considering the railway maintenance environment. In conclusion, considering the railway vehicle maintenance environment and device specifications, it was inferred that the relatively simple and lightweight LeNet-5 model can be effectively utilized while using small images.

## 1. Introduction

Research related to CBM (condition monitoring maintenance) and PHM (prognostics health and management) is being conducted worldwide in the railway sector. In the case of the Republic of Korea, a significant amount of research related to smart maintenance is being carried out as a part of a national R&D project due to the rapid decrease in population. Currently, the preventive maintenance method is the most commonly used in the field of railway vehicles [1]. In addition, during the stage of light maintenance prior to heavy maintenance, faults are often visually inspected by the mechanics themselves. As a result, there are instances where indicators of potential failures are overlooked, or defects remain undetected. Furthermore, a disadvantage exists in that the preventive maintenance method requires the premature replacement of parts that are still available. For this reason, maintenance costs increase. Therefore, it was determined that applying CBM technology to the current maintenance method, preventive maintenance could reduce maintenance costs. For example, in the case of wheels of railway vehicles, which is the subject of this paper’s research, wheel turning is required according to the safety standard of Table 1 in the Republic of Korea data from [2]. However, due to mechanics’ work errors, wheel turning is frequently performed, even when it is not necessary. Therefore, this suggests that the introduction of CBM technology could reduce unnecessary maintenance costs. In addition, in the case of Korea, the railway vehicle maintenance market is about USD 400 million. Of these, light maintenance is USD 280 million, accounting for about 70% [3]. For this reason, this indicates that the railway sector could develop further if it invested elsewhere by reducing unnecessary maintenance costs.

Chenyi Zhou et al. proposed a method of detecting the location of wheel flats by installing sensors on rails. At this time, a method of finding a location using the response results through the rail strain was proposed through a sensor installed on the rail [4]. Stasys Steišūnas et al. modeled the railway vehicle and wheel flats using SIMPACK, a multi-body dynamics software. They also proposed a method to define the parameters of the suspension using acceleration data generated by wheel flats [5]. Xinyu Peng et al. conducted research by modeling the railway vehicle and wheel through SIMPACK, as in this paper and Stasys Steišūnas’ papers. At this time, Xinyu Peng compared and verified the SIMPACK model and the data of the actual railway vehicle. As such, in the field of railways, many researchers, including this paper, use SIMPACK, a dynamics software. Therefore, it is possible to prove the reliability of SIMPACK through many papers [6]. Bo Liang et al. conducted research to detect surface defects on wheels and rails. They applied STFT (short-time Fourier transform), WVT (Wigner–Ville transform), and WT (wavelet transform) for their study [7]. Mohammadreza Mohammadi et al. used unsupervised learning to detect wheel flats. At this time, the data were obtained through 113 simulations without using an actual vehicle. Also, the data were obtained from the rail, not from the railway vehicles. In addition, a total of four unsupervised learning models were used [8]. Chuncheng Yang et al. used supervised learning methods to detect defects in rails. Unlike this paper, a wavelet was selected for signal processing at this time. And the deep learning models used were ResNet and FCN [9]. Long Zhang et al. proposed a method for diagnosing defects in a rotating machine by applying a CNN algorithm. At this time, the model was evaluated by proposing an improved model based on the LeNet-5 model, similar to this paper [10]. Run Gao et al. proposed a method of detecting wheel flats using a reflective optical position sensor. The research was conducted by making a multi-body dynamics model. At this time, unlike this paper, sensors were installed on a rail, not a wheel [11]. Gabriel Krummenacher et al. proposed two methods to detect anomalies in wheel flats. First, unlike this paper, they used the SVM (support vector machine) model based on wavelets to detect wheel flats in time series data. Second, they used the deep neural network (DNN) algorithm to detect wheel flats. The results of the two methods were then compared and analyzed [12]. Yunguang Ye et al. first discussed the severity of shocks resulting from faults in rotating machinery. Subsequently, they conducted research on shock detection to diagnose these faults. They proposed an adaptive feature called ATDI (activated time–domain image) and utilized a hybrid approach, ATDI-NN, combining DNN for wheel flat detection [13]. Yunguang Ye et al. proposed a novel entropy called MTFIE (multislice time-frequency image entropy) for wheel fault diagnosis. They utilized time-frequency images incorporating machine fault information [14]. J. Brizuela et al. introduced the use of ultrasound techniques for wheel flat detection. They employed Rayleigh wave ultrasonic pulses for detection. Finally, they successfully validated the detection of wheel flats using this technology [15]. Araliya Mosleh et al. utilized envelope spectrum analysis for the detection of wheel flats. They tested and analyzed the sensitivity of the proposed method. Finally, they concluded that it is an efficient approach for wheel flat detection [16]. Esteban Bernal et al. presented a dynamic verification of an onboard wheel flat detection technique using analog signal processing. And this was shown to reduce the power consumption and hardware costs of condition-monitoring sensor nodes [17]. Yunguang Ye et al. conducted research that considered not only the detection of wheel flats but also the estimation of wheel flat length. They utilized a dynamics model for simulation-based research. They constructed a KSM (Kriging surrogate model) and applied a PSO (particle swarm optimization)-based algorithm. Furthermore, they verified their findings through field tests, ultimately demonstrating the ability to estimate wheel flat length [18]. Yunguang Ye et al. investigated the induction and exacerbation of wheel polygonization caused by wheel flats. They also examined the impact of the speed of railway vehicles and the length of wheel flats on wheel polygonization. This was not within the scope of the research direction and objectives of our paper. However, it is considered a valuable resource for future research on wheel flats [19]. Dachuan Shi et al. proposed a machine fault diagnostic model in the railway domain through data augmentation. They introduced a data augmentation framework called MBS-FWFSA (multibody dynamic simulation-fast-weighted feature-space averaging) and used it to enhance the robustness of machine-learning-based fault diagnosis methods as proven in their study [20].

In this paper, further research was conducted based on the knowledge that the researchers of this paper learned through prior research. First of all, in the prior paper, vibration signals caused by wheel flats were measured, and raw data and signal-processed data were used for learning, leading to a comparison of accuracy [21]. In this paper, to differentiate it from the related studies mentioned above, consideration was given not only to simple wheel flat anomaly detection but also to the number and size of wheel flats. In fact, not only the size of wheel flats but also the number of wheel flats and the wheel diameter affect the vibrated acceleration signal. Therefore, we conducted the research, taking all of these aspects into consideration. At this point, the research conducted anomaly detection by distinguishing between anomaly data and normal data based on the fault regulations outlined in Table 1. Next, the signal-processed data were compared through the supervised and semi-supervised methods. The reason for this comparison is written in Section 4. And, in this paper, deep learning was based on CNN (convolution neural network). Subsequently, the results of the deep learning process were evaluated by means of accuracy, recall, and the ROC curve.

## 2. Modeling and Wheel Flats Signal

### 2.1. Wheel Flats

**Figure 1:**
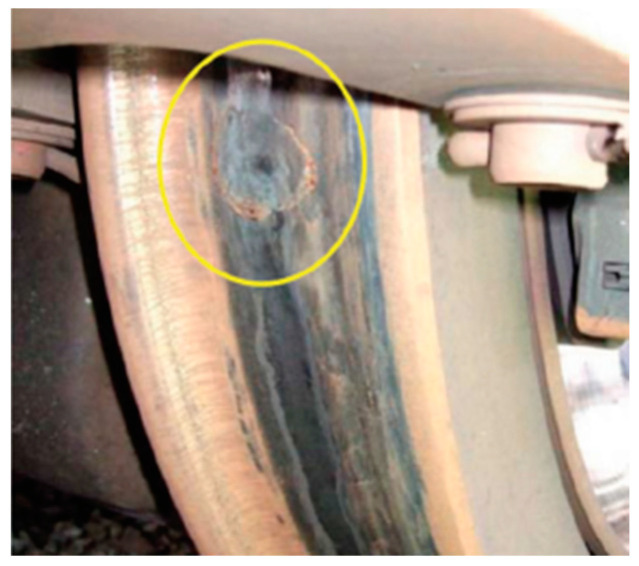
Wheel flat [22].

In this paper, a study on the condition monitoring (anomaly detection) of wheel flats was conducted. As shown in Figure 1, wheel flats occur due to imbalanced friction or impacts at the contact area between the wheel and the rail. Therefore, whenever the wheel contacts the rail, vibration acceleration signals resulting from impacts can be observed. These vibrations have an impact on ride quality degradation and operational safety.

**Figure 2:**
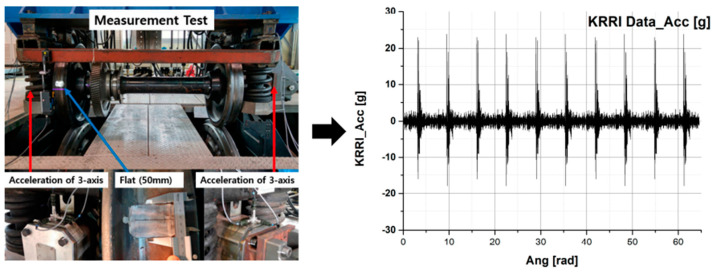
Measurement test using a roller rig at the KRRI [23].

Indeed, the vibration acceleration signal by wheel flats could be confirmed, as shown in Figure 2, through other studies in the past. Figure 2 is the result of checking what vibration acceleration signal appears when wheel flats occur using a roller rig at the KRRI (Korea Railroad Research Institute). At that time, the wheel flat test was conducted at a constant velocity of 20 km/h, with a 50 mm flat on the left wheel.

### 2.2. Reasons for Using a Multi-Body Dynamics Model

In this paper, SIMPACK, a multi-body dynamics software, was used to conduct the research. There are two main reasons that we cannot test using actual railway vehicles. First, this paper conducted deep learning utilizing the acceleration induced by vibration when wheel flats occur. Thus, it is a study that requires a substantial amount of big data. For example, there are various cases such as the number, size, and location of wheel flats when wheel flats occur. It is very difficult to test the number of these various cases on actual railway vehicles. The creation of intentionally induced wheel flats on real wheels demands substantial time and money to cover all potential scenarios. In this paper, it was research on anomaly detection methods on the scale of a university research institute. Therefore, the best way to validate the phenomenon was to simulate the number of various cases using the dynamics model rather than using actual railway vehicles.

Second, the railway sector comes within the public industries where safety and service are important. For this reason, it is very difficult to conduct the test on the actual operating track. For example, testing broken railway vehicles can lead to an accident. And researchers cannot test on the track during operating hours due to the passengers. Therefore, it is only available for three to four hours at dawn. Of course, there is one test track section in Korea. However, researchers have to pay about USD 7500 a day to use this test track. Moreover, it is very difficult to make a reservation to use it because there is only one place for testing in the Republic of Korea. Therefore, in order to analyze detection such as in this study, it is not possible to conduct tests using actual faulty trains. So, many studies and papers in the field of railway vehicles replace it through simulation.

### 2.3. Multi-Body Dynamics Modeling of Railway Vehicles and Wheel Flats

**Figure 3:**
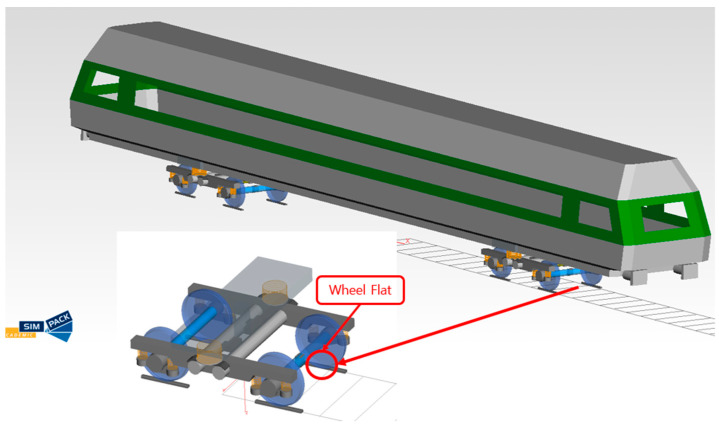
Modeling of railway vehicles and wheel flats.

Figure 3 is a railway vehicle model made using SIMPACK, a multi-body dynamics software [24]. In this model, the vehicle and bogie were modeled based on the specifications of actual operating vehicles from ‘SeoulMetro’, one of the railway operation companies in the Republic of Korea. Other modeling details include a track gauge of 1435 mm standard gauge. Additionally, the rail type was UIC 60 rail, and the wheel-rail interface applied the Kalker contact theory, using the SIMPACK rail module.

**Figure 4:**
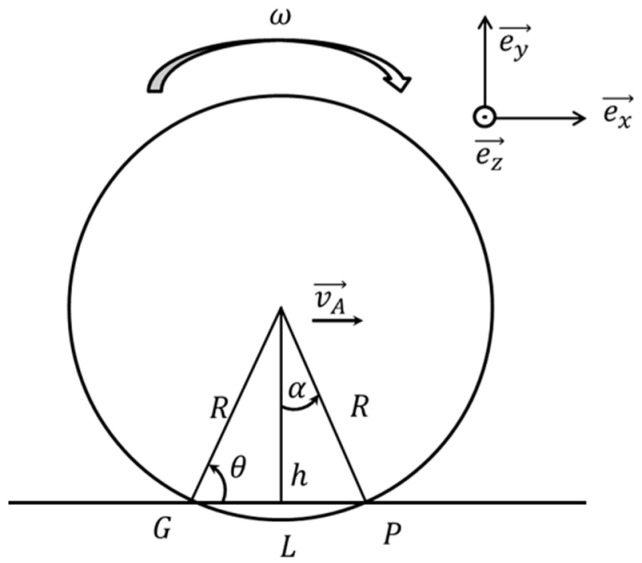
Calculating coordinates for wheel flat configuration [25].

Wheel flats were modeled in one wheel, as shown in Figure 3 above. At this time, wheel flats were simulated with various sizes, various numbers, and various locations. In addition, in order to model wheel flats, coordinate values were obtained by constructing formulas, as shown in Equations (1) through (4) based on Figure 4. The obtained coordinate values were input to model the wheel with wheel flats [25].

(1) $$\theta \;=\;\cos^{-1}\frac{L/2}{R}$$

(2) $$h=\;\frac{L}{2}tan$$

(3) $$cos\alpha =\frac{h}{R}$$

(4) $$\alpha =\cos^{-1}\frac{h}{R}$$

Table 2 shows some cases of modeling. In this paper, a total of 4977 cases were modeled by varying the diameter of the wheel, and the number and position of wheel flats with the same configuration as in the example of Table 2. At this time, the size and number of wheel flats were modeled by referring to the safety standard in Table 1. Additionally, the reason for considering not only the number and size of wheel flats but also the diameter of the wheels was that the magnitude of vibrated acceleration varies depending on the wheel’s diameter.

**Figure 5:**
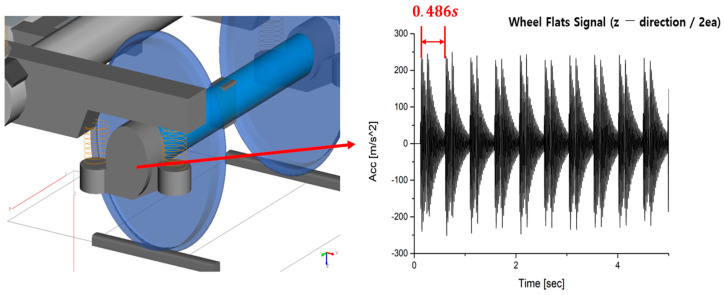
The results of the simulation using SIMPACK Software.

As described above, Figure 5 shows the results of the simulation using SIMPACK Software. At this time, the simulation was conducted at a constant velocity of 20 km/h. These are the results for the case of having two occurrences of wheel flats. Through Figure 5, it is confirmed that signals resulting from wheel flats occurred effectively at intervals of 0.486 s (velocity: 20 km/h). In addition, as observed in the results of the roller rig test, the magnitude of vibrations caused by wheel flats was accurately represented. Based on these simulation results, deep learning will be learned through the learning data production process outlined in Section 3.

## 3. Learning Data Production Process

### 3.1. Overlapped Noise and Simulation Signals

**Figure 6:**
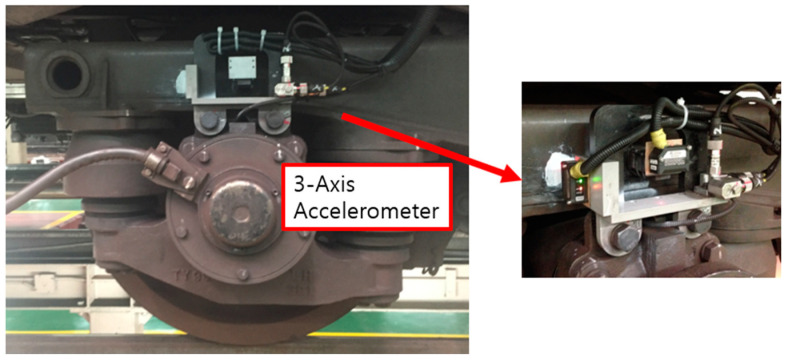
Location of the equipment for measuring vibration acceleration [26].

Prior to Section 3, Section 2 of this paper explained the reason for obtaining data through dynamics software rather than actual vehicles. However, the disadvantage of a simulation is that noise signals are not properly included. Therefore, in this paper, noise signals obtained from actual-driving railway vehicles through national R&D in the past were used. At that time, induced acceleration data were measured using a three-axis accelerometer, as shown in Figure 6 data from [26]. In addition, in order to utilize the acceleration data measured through actual railway vehicles, the dynamics model of this paper modeled railway vehicles of the same manufacturer and on the same route as the actual railway vehicles.

**Figure 7:**
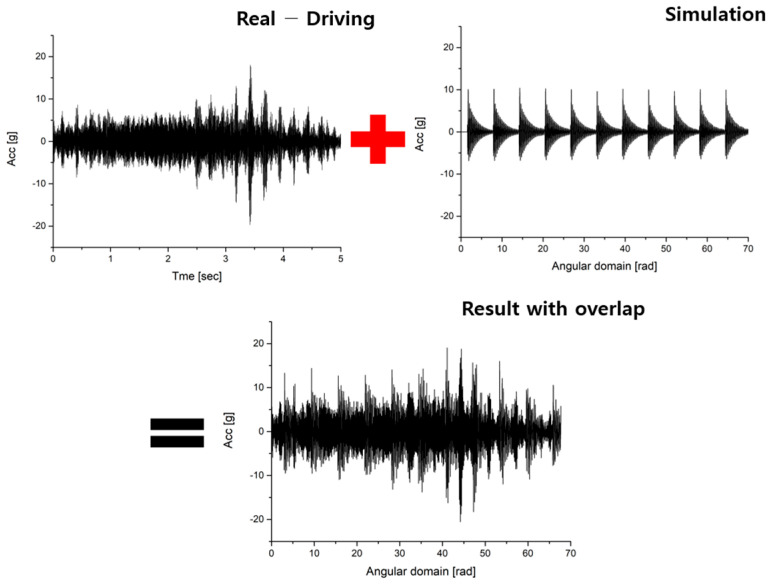
Overlapping result of real driving and simulation (vibration acceleration).

The noise signal overlapping the simulation signal used data from the section with the most severe noise among the measured data. The reason for selecting the segment with the most intense noise was to consider unfavorable conditions. Figure 7 shows the overlapping results.

### 3.2. Signal Processing for Application to Deep Learning

**Figure 8:**
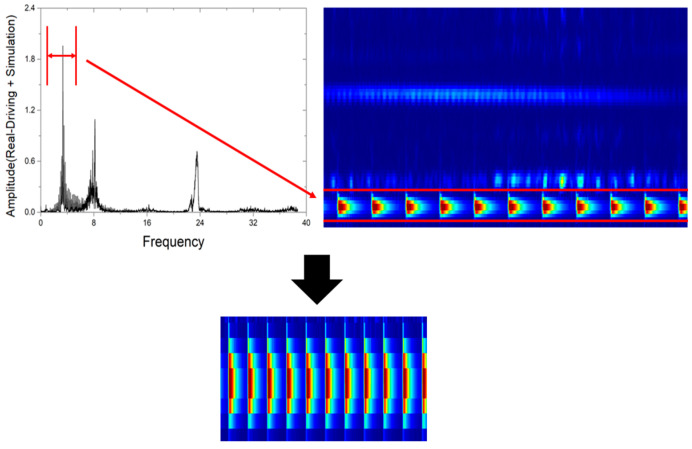
FFT for frequency band verification by wheel flats.

As shown in Figure 7, signal processing was conducted to enable the utilization of overlapped signals in CNN-based deep learning. In this process, STFT (short time Fourier transform) was selected for signal processing. First of all, order analysis was used in the time domain. In this paper, order analysis is defined as the number of events occurring per unit rotation. Order analysis requires changing the signal to the angular domain before performing FFT of the time domain data [27]. Next, the signal converted into the angular domain was subjected to fast Fourier transform (FFT) to identify the frequency band affected by wheel flats. Then, as shown in Figure 8, a band-pass filter was utilized to isolate the frequency band affected by wheel flats, and STFT was performed. And through this, spectrogram images were obtained. At this time, the parameter values for STFT are shown in Table 3 [21].

**Figure 9:**
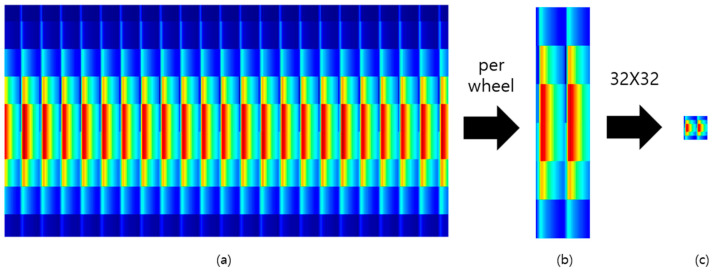
Spectrogram image cropping and resizing for LeNet-5 and ResNet-20. [(**a**): Raw Image (**b**): Per wheel (**c**): 32X32 image].

Finally, as shown in Figure 9, the image size was reduced to 32X32 in order to utilize the selected LeNet-5 and ResNet-20 models in this paper. At this time, only the x-axis range corresponding to one rotation was cropped and utilized. Figure 9 represents the case where there are two instances of wheel flats.

**Figure 10:**
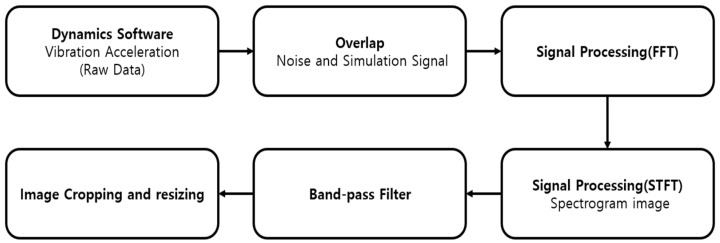
Signal processing process for CNN (convolution neural network).

And Figure 10 is a diagram of the entire process.

**Figure 11:**
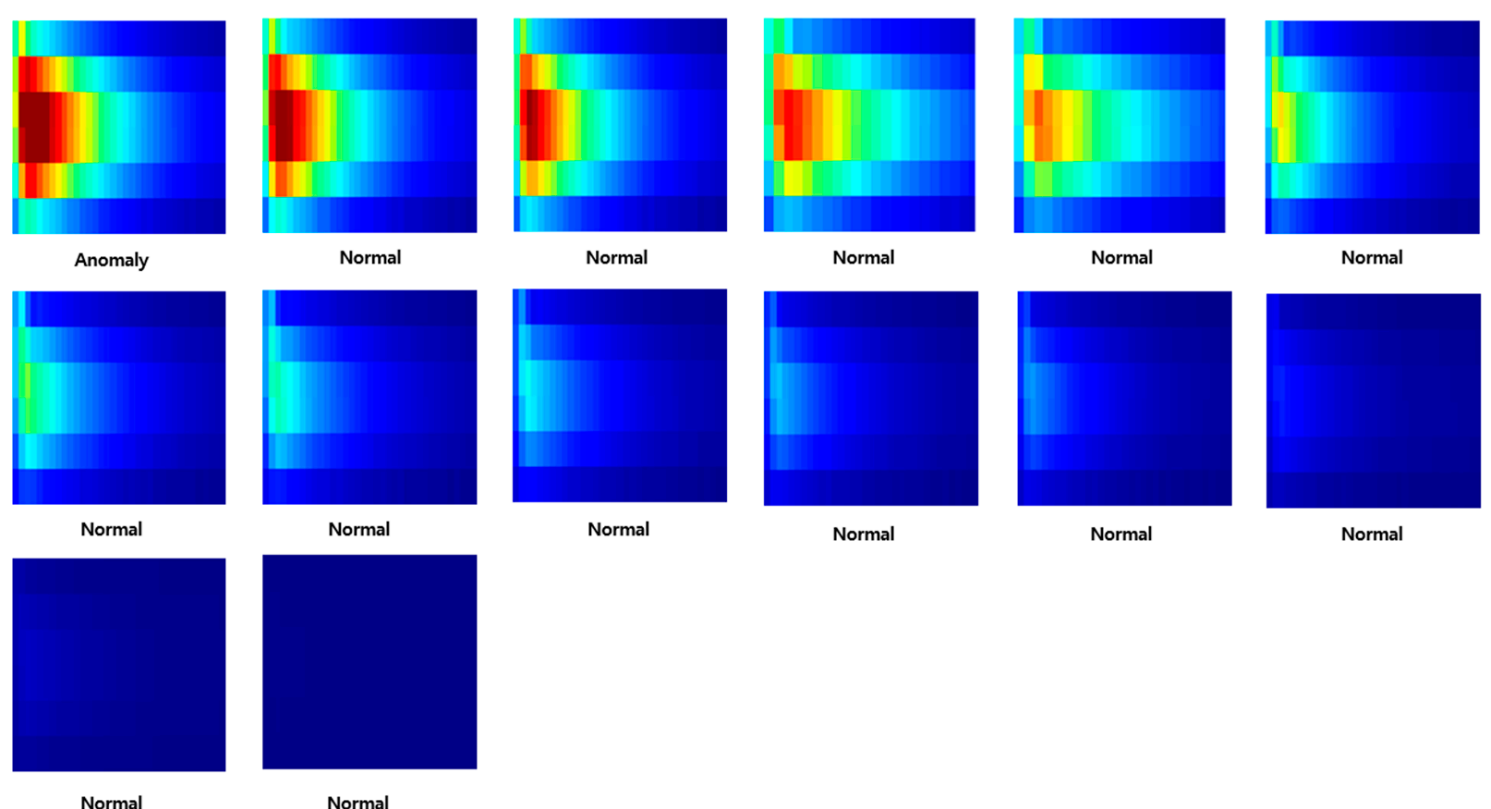
Samples of anomaly and normal data (wheel flat: 1ea).

**Figure 12:**
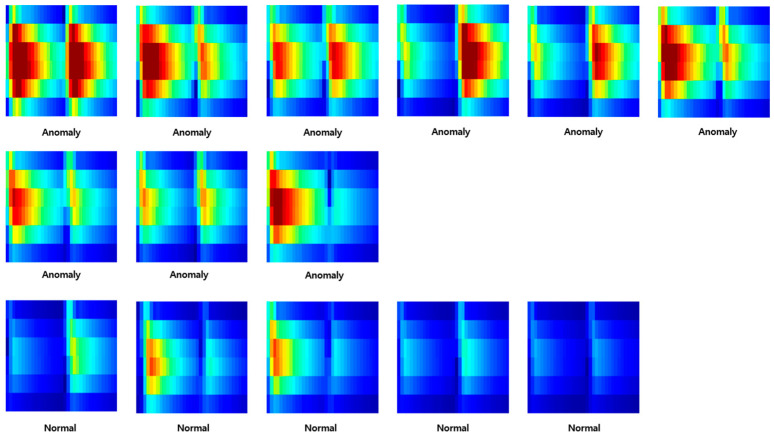
Samples of anomaly and normal data (wheel flat: 2ea).

**Figure 13:**
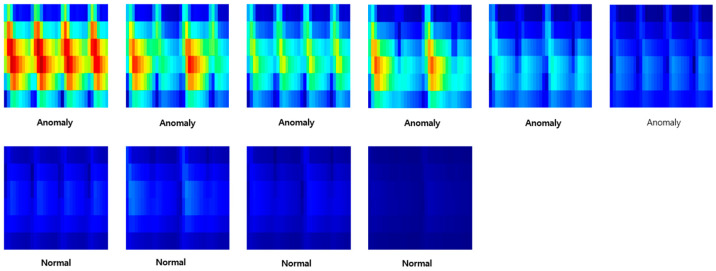
Samples of anomaly and normal data (wheel flat: 4ea).

Through this entire process, it was finally possible to construct data for deep learning. At this time, both anomaly data and normal data were classified based on Table 1 in the introduction part. Figure 11, Figure 12 and Figure 13 are the anomaly and normal data (spectrogram image) for cases with one, two, and four instances of wheel flats. These figures show some cases, and the total number of images used for learning was 4977.

The data of 4977 were composed of the dataset as shown in Table 4. In this case, the dataset generally consists of 60% for training data, 20% for validation data, and 20% for test data [28].

## 4. Comparison of Deep Learning Results between Supervised and Semi-Supervised Learning

### 4.1. Supervised Learning Method for Anomaly Detection in Railway

#### 4.1.1. Modified the LeNet-5 (Supervised Learning)

**Figure 14:**
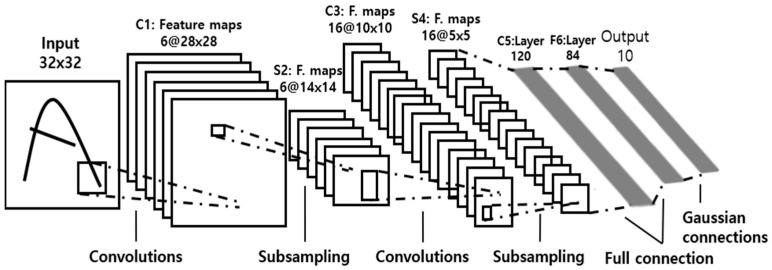
Architecture of LeNet-5 [29].

First of all, in this paper, the obtained spectrogram images through the processes of Section 2 and Section 3 were utilized for conducting research based on CNN. First, referring to the LeNet-5 model, the activation function and pooling settings were changed to improve speed and performance in the existing architecture. LeNet-5 is a model created in 1998 by Yann LeCun, who first proposed the CNN concept data from [29]. Figure 14 shows the architecture of LeNet-5. At this time, the existing LeNet-5 used the sigmoid function and average pooling in the convolution and subsampling process. However, in this paper, the sigmoid function was changed to Leaky ReLU. And average pooling was changed to max pooling.

#### 4.1.2. ResNet-20 (Supervised Learning)

**Figure 15:**
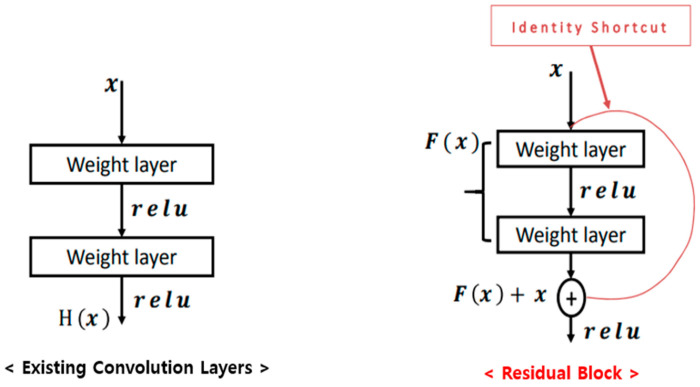
The structure of existing convolution layers and residual block [30].

Second, this paper used the ResNet-20 model. First of all, ResNet was introduced in a paper called ‘Deep Residual Learning for Image Recognition’ in 2015 [30]. The ResNet is a model with the concept of residual block using skip connection, as its name suggests [30]. Figure 15 and Equations (5)–(12) show the differences from the existing convolution layers. At this time, Equations (5)–(8) represent the existing process, and Equations (9)–(12) represent the process with applied residuals.

<Without Skip Connection>

(5) $$\mathrm{Liner}\;\mathrm{Layer}\;1:\;z^{\left[l+1\right]}\;=\;W^{\left[l+1\right]}a^{\left[l\right]}+b^{\left[l+1\right]}$$

(6) $$\mathrm{ReLU}\;\mathrm{operation}\;\mathrm{on}\;\mathrm{Linear}\;\mathrm{Layer}:\;a^{\left[l+1\right]}=g\left(z^{\left[l+1\right]}\right)$$

(7) $$\mathrm{Linear}\;\mathrm{Layer}\;2:\;z^{\left[l+2\right]}\;=\;W^{\left[l+2\right]}a^{\left[l+1\right]}+b^{\left[l+2\right]}$$

(8) $$\mathrm{ReLU}\;\mathrm{operation}\;\mathrm{on}\;\mathrm{Linear}\;\mathrm{Layer}\;2:\;a^{\left[l+2\right]}=g\left(z^{\left[l+2\right]}\right)$$

<With Skip Connection>

(9) $$\mathrm{Liner}\;\mathrm{Layer}\;1:\;z^{\left[l+1\right]}\;=\;W^{\left[l+1\right]}a^{\left[l\right]}+b^{\left[l+1\right]}$$

(10) $$\mathrm{ReLU}\;\mathrm{operation}\;\mathrm{on}\;\mathrm{Linear}\;\mathrm{Layer}:\;a^{\left[l+1\right]}=g\left(z^{\left[l+1\right]}\right)$$

(11) $$\mathrm{Linear}\;\mathrm{Layer}\;2:\;z^{\left[l+2\right]}=\;W^{\left[l+2\right]}a^{\left[l+1\right]}+b^{\left[l+2\right]}$$

(12) $$\mathrm{ReLU}\;\mathrm{operation}\;\mathrm{on}\;\mathrm{Linear}\;\mathrm{Layer}\;2:\;a^{\left[l+2\right]}=g\left(z^{\left[l+2\right]}+a^{\left[l\right]}\right)$$

The first reason for choosing ResNet-20 in this paper was the image size of the learning data. In the process outlined in Section 2 and Section 3, the learning data were processed from the spectrogram image to the image size of 32 × 32. Therefore, ResNet-20 was selected to standardize the image size of the models used in this paper’s research. The second reason was that the majority of employees of railway operators lack knowledge related to deep learning. Therefore, it was chosen to enhance accessibility for non-experts. The reason was that LeNet-5 and ResNet-20 are not the latest models, but they are representative image classification models on CNN. And they feature a relatively concise and comprehensible structure. The third reason was that relatively lighter models were selected in consideration of this part, as problems with memory and performance (communication, device specifications, etc.) of devices used in the railway environment may also occur.

#### 4.1.3. MobileNet-V3 (Supervised Learning)

**Figure 16:**
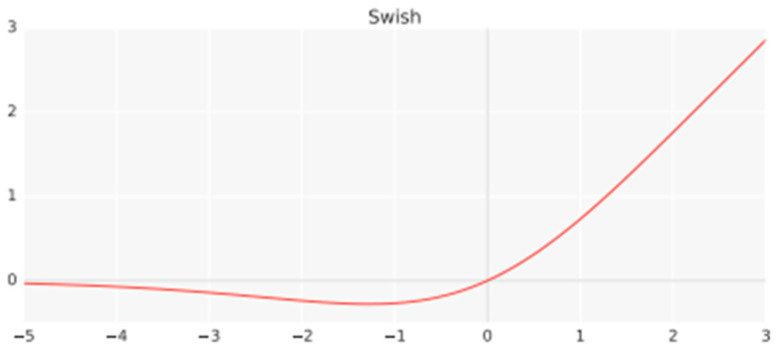
Swish activation function [30].

Third, the MobileNet-V3 model was utilized. MobileNet-V3 was initially introduced through the paper ‘Searching for MobileNetV3’ in 2019 [31]. The paper discusses the development process and performance of MobileNet-V3, a lightweight deep learning model. MobileNet-V3 primarily focuses on reducing the number of parameters and FLOPs compared to its predecessors, MobieNet-V1 and V2. This emphasis enables model lightweight and faster computations. Unlike previous MobileNet models, MobileNet-V3 employs the swish activation function, as shown in Figure 16 with data from [32].

Therefore, in this paper, the research was conducted by adding the latest model, MobileNet-V3. In the case of the original MobileNet-V3, it utilizes 224 × 224 images. However, in this paper, modifications were made to enable the use of 32 × 32 images. In other words, MobileNet-V3 was customized to be suitable for the CIFAR dataset, allowing it to use the same image size as the LeNet-5 and ResNet-20 models.

Ultimately, railway vehicle devices may encounter issues related to memory and performance limitations, such as communication problems and CPU constraints. Therefore, lighter weight and more concise models were chosen, considering these factors.

### 4.2. Semi-Supervised Learning Method for Anomaly Detection in Railway

#### Deep One Class Classification

In this paper, besides the supervised learning using LeNet-5 and ResNet-20, a reference was made to a paper on semi-supervised learning using the Deep One Class Classification. The reason is that in the case of the railway sector, the preventive maintenance method is currently the most used. For example, when railway vehicles come in after operation, the light maintenance department performs daily and monthly inspections. In addition, the heavy maintenance department performs intermediate (every 3 years) and comprehensive (every 6 years) inspections. Therefore, due to the regular replacement or maintenance of parts before malfunctions occur, it is difficult to obtain faulty data instead of normal data. For this reason, in order to utilize only normal data, semi-supervised learning was also used in addition to supervised learning.

**Figure 17:**
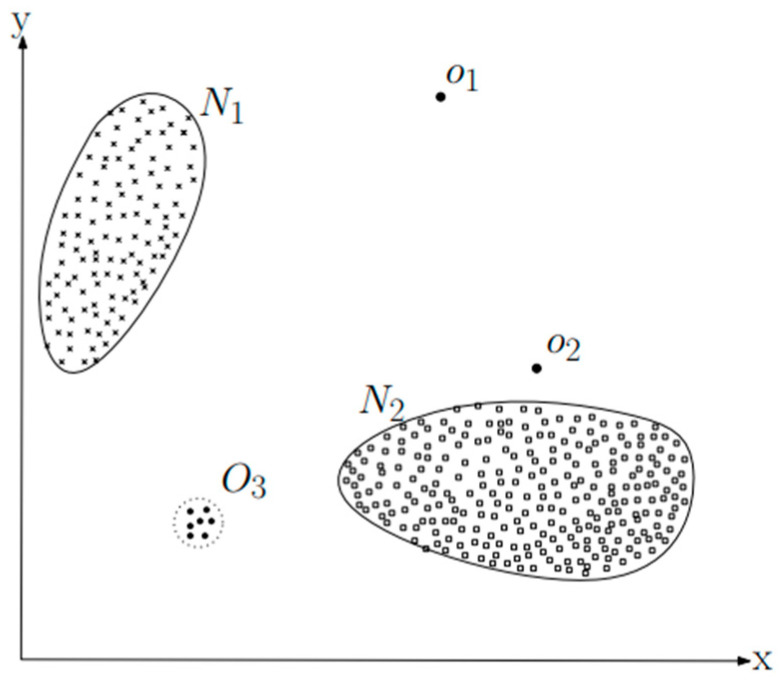
A simple example of point anomaly (two regions) [33].

**Figure 18:**
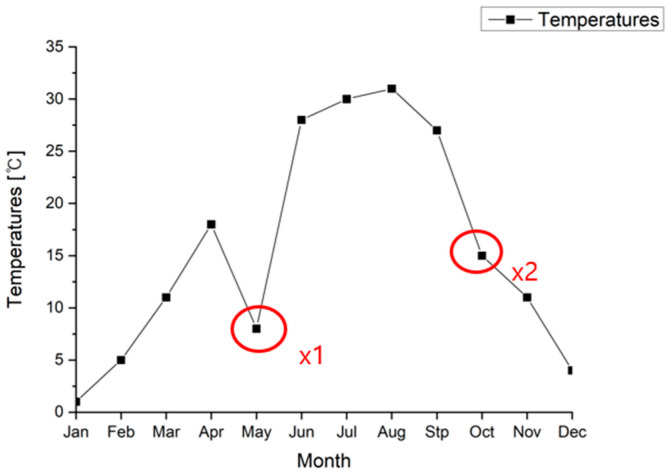
A simple example of contextual anomaly (time-series/temperature).

First of all, in anomaly detection, there are concepts of point anomaly and contextual anomaly. Figure 17 and Figure 18 show examples of point analysis and contextual analysis data from [33].

For example, in time-series data, as shown in Figure 18, sudden temperature changes like x1 and x2 can lead to anomaly detection. Next, as shown in Figure 16, anomaly data such as Q1, Q2, and Q3 outside the two normal regions (N1, N2) can be detected [24].

Here, Deep One Class Classification is one of the concepts of point analysis detection as shown in Figure 17. First of all, Deep One Class Classification was announced at the ICML (International Conference on Machine Learning) in 2018. It is an approach that combines deep learning with the traditional machine-learning-based one class classification method, SVDD (support vector data description). While the conventional SVDD was kernel based, Deep SVDD learns the feature space through deep learning and seeks the optimal hypersphere that encloses normal data within that feature space. At this point, the objective function of the conventional SVDD is represented as Equation (13), and the objective function of Deep SVDD is expressed as Equation (14) [34].

(13) R2R, c, ξmin+1vn∑iξi

(14) R2R, Wmin+1vn∑i=1nmax{0,∅(xi;W)−c2−R2}+λ2∑i=1nWlF2−R2}

**Figure 19:**
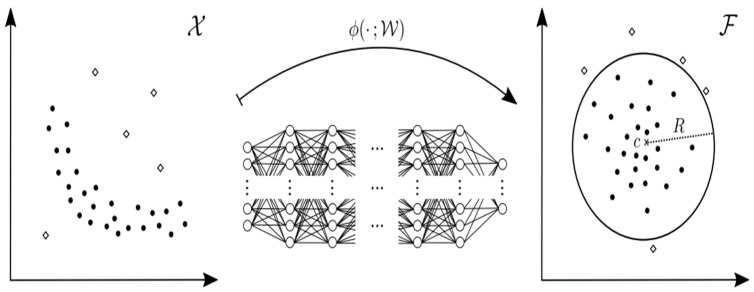
Architecture of Deep SVDD [34].

In conclusion, Deep SVDD extracts the feature space of normal data through the objective function in Equation (14) above. Then, the points of each data are mapped close to the center c of the sphere to find the optimal hypersphere. Figure 19 shows the architecture of Deep SVDD data from [34].

### 4.3. Evaluation Methods for the Results of Deep Learning in Supervised and Semi-Supervised Learning

#### 4.3.1. Comparison Results of LeNet-5 and ResNet-20

The results of this paper study were evaluated through accuracy, recall, and ROC (receiver operating characteristic) curve. First, the true positive (TP), true negative (TN), false positive (FP), and false negative (FN) were obtained through the confusion matrix. Through this, accuracy, precision, recall, and F1 score can be obtained. Here, precision means how many of the things classified as failures are actually failures. Recall means how many values are properly classified as failures in the actual failure data. Therefore, the importance of precision and recall varies depending on the purpose of the evaluation [35].

For the purpose of this paper, recall was selected because it is more important than classifying normal as anomalies, as accurately identifying anomalies as anomalies. Equations (15)–(18) are the methods of obtaining these [24]:

(15) $$Accuracy=\frac{TP+TN}{TP+FP+TN+FN}$$

(16) $$Precision=\frac{TP}{TP+FP}$$

(17) $$Recall=\frac{TP}{TP+FN}$$

(18) $$F1\;Score=\frac{2TP}{2TP+FP}$$

Next, the evaluation was conducted by calculating the AUC (area under the curve) through the ROC curve. The ROC curve is an evaluation method used when recall is important [36]. Additionally, the ROC curve and AUC are primarily employed in binary classification. Since this paper study is a binary classification that distinguishes between normal and failure, ROC curve and AUC were selected [37].

**Figure 20:**
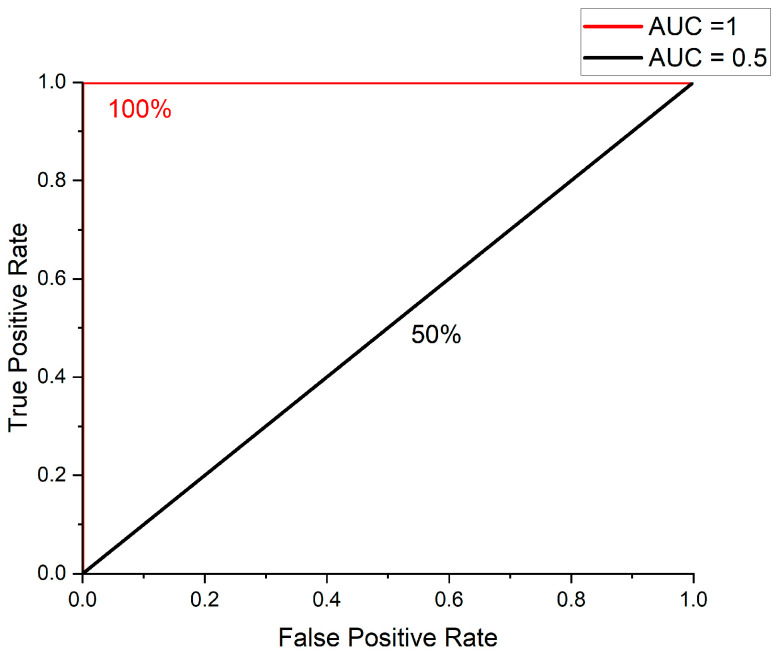
ROC curve and AUC plotting example.

The ROC curve is plotted using the values of TPR (true positive rate) and FPR (false positive rate) [38]. Figure 20 shows the ROC curve. Here, TPR is represented on the y-axis, and FPR is on the x-axis. In the case of AUC obtained through ROC curve, it has a value between 0 and 1. At this time, if it is close to 1, it means that the classification performance is excellent, and if it is close to 0.5, it is judged that it is similar to the random prediction [39].

#### 4.3.2. Comparison Results of Modified LeNet-5 and ResNet-20

**Figure 21:**
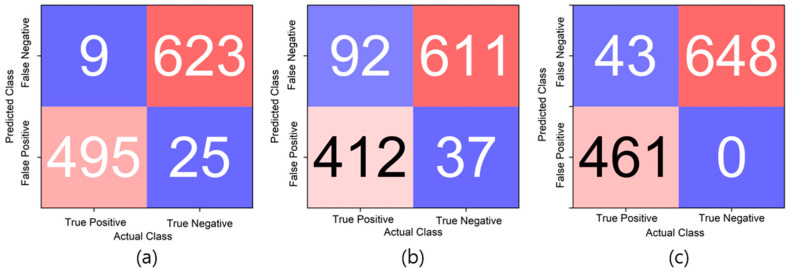
Confusion matrix results for modified LeNet-5 and ResNet-20 model ((**a**) modified LeNet-5/(**b**) ResNet-20/(**c**) modified MobileNet-V3).

**Figure 22:**
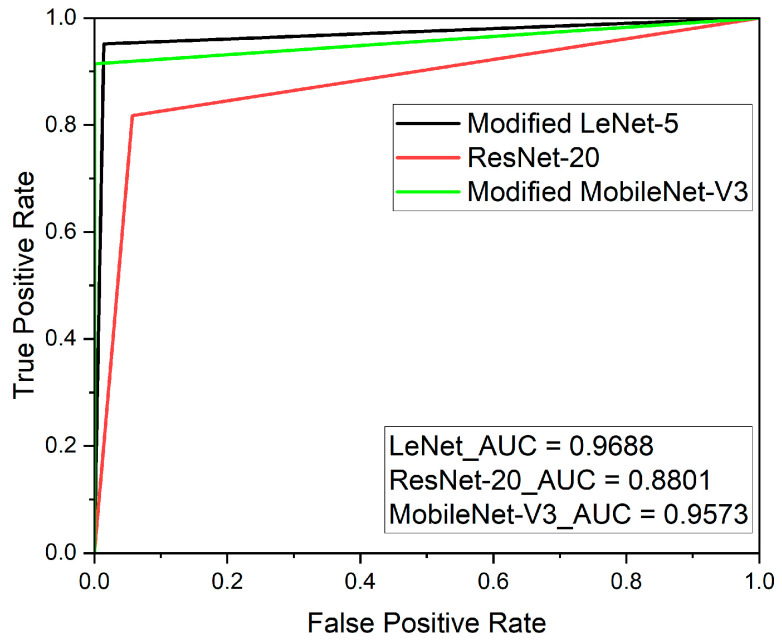
ROC curve results for the modified LeNet-5, ResNet-20 model, and modified MobileNet-V3 models.

The deep learning results of the modified LeNet-5, ResNet-20, and modified MobileNet-V3 models have confusion matrices, as shown in Figure 21. The accuracy values were approximately 97%, approximately 89%, and approximately 96%, respectively. The recall scores were approximately 0.982, approximately 0.817, and approximately 0.915, respectively. Finally, the AUC values obtained through the ROC curve were approximately 0.9688, approximately 0.8801, and approximately 0.9573, respectively, as shown in Figure 22. The overall results are presented in Table 5. At this time, the results were not evaluated based on the presence or absence of wheel flats but rather evaluated based on the distinction between anomaly data and normal data according to the regulations in Table 1.

Overall, it was confirmed that the modified LeNet-5 and MobileNet-V3 models produced outstanding results.

#### 4.3.3. Comparison Results of Supervised and Semi-Supervised Learning

**Figure 23:**
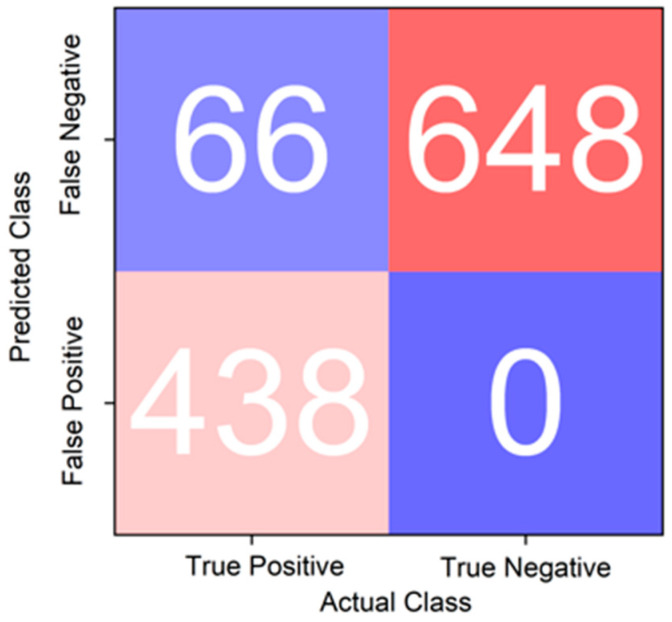
Confusion matrix results for Deep One Class Classification (Deep SVDD).

**Figure 24:**
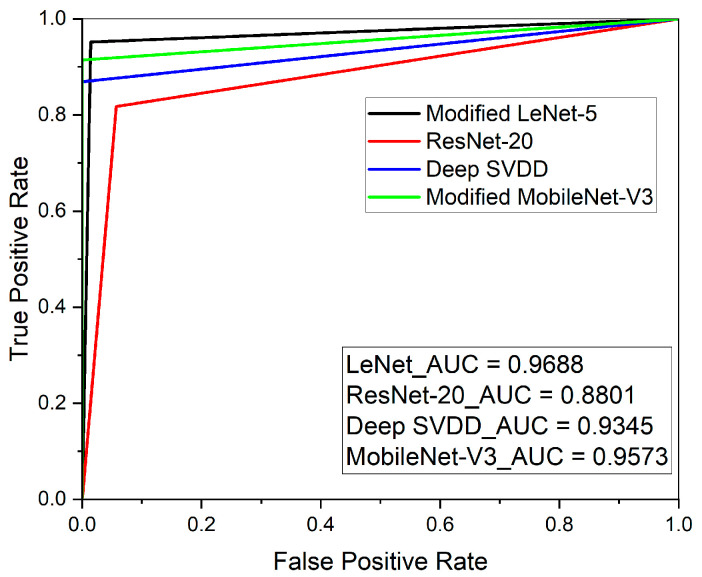
ROC curve results for supervised and semi-supervised learning.

The deep learning results of the supervised and semi-supervised learning methods are as follows. In this case, the order of results was modified LeNet-5, ResNet-20, modified MobileNet-V3, and Deep One Class Classification (Deep SVDD). First of all, the deep learning result of the semi-supervised learning had a confusion matrix, as shown in Figure 23. The accuracy values were approximately 97%, approximately 89%, approximately 96%, and approximately 94%, respectively. The recall scores were approximately 0.982, approximately 0.817, approximately 0.915, and approximately 0.869, respectively. Lastly, the AUC values obtained through the ROC curve were approximately 0.9688, approximately 0.8801, approximately 0.9573, and approximately 0.9345, respectively, as shown in Figure 24. The overall results are presented in Table 6.

Overall, it was confirmed that the supervised method using the modified LeNet-5 and modified MobileNet-V3 models had better anomaly detection results for wheel flats compared to the Deep One Class Classification (Deep SVDD) model. However, it was confirmed that the semi-supervised Deep One Class Classification (Deep SVDD) model also had a high accuracy value. Therefore, it was judged that this result was a significant study in an environment where it was difficult to learn failure data due to the repair method of the railway sector, which is preventive maintenance.

## 5. Conclusions

In this study, additional studies were conducted based on the research results preceded by the authors of this paper. In this paper, the signal-processed data were used and analyzed by selecting CNN (convolution neural network)-based models in consideration of the railway environment. At this time, the analysis of the results was conducted using accuracy, recall, and AUC.

First, for the purpose of anomaly detection of wheel flats in this paper, the multi-body dynamics software SIMPACK was utilized. In addition, the obtained acceleration through simulation results was overlapped with the vibration acceleration of actual driving railway vehicles. At this time, the vibrated acceleration was set to the section with the highest noise. Finally, the result of overlapping the simulation signal results and the actual signal results was signal-processed. At this time, STFT was selected for signal processing to be applied to the CNN algorithm. Spectrogram images obtained through STFT were used for learning.

Second, most employees of railway operators lack knowledge related to deep learning. Current railway vehicles perform control, monitoring, and communication through the TCMS (train control and management system). These vehicles do not have devices such as GPUs (graphics processing units), which support complex analysis and data processing. Therefore, models with relatively simple structures and lightweight characteristics were chosen.

Third, the supervision and the semi-supervision learning methods were compared and analyzed. Currently, the preventive maintenance method is the most commonly used in the field of railway vehicles. For this reason, it is not easy to obtain failure data. In this paper, the semi-supervised learning method was also conducted in consideration of these points.

Finally, as a result of the analysis, the modified LeNet-5 model and the modified MobileNet-V3 model achieved approximately 97% and 96% accuracy, respectively. And the modified LeNet-5 model and modified MobileNet-V3 model had inference times of 782 ms and 766 ms, respectively. In other words, considering the requirement of the real-time performance of 1 s on currently working railway vehicles that do not have a GPU, it was evident that choosing relatively simpler and lightweight models met the requirement. Next, although the semi-supervised learning results were not better than the modified LeNet-5 model and modified MobileNet-V3, they showed significant results considering the railway maintenance environment. Ultimately, despite being an older model than MobileNet-V3, based on these results, it was concluded that the LeNet model is also suitable for application when considering the device specifications of railway vehicles.

For the development of future research, the results will propose a plan at a national R&D rather than a university research institute scale. Through this, this paper’s research method will be applied to actual railway vehicles to develop in consideration of device specifications (computer specifications) of railway vehicles, safety problems when installing sensors, and data communication problems.

## Figures and Tables

**Table 1:** Safety standard for domestic urban railway vehicles [2].

The Number of Wheel Flats (EA)	Flat Size (mm)
1	75 < Flat size
2	50 ≤ Flat size < 75
4	25 ≤ Flat size < 50

**Table 2:** Example of wheel flat configurations (in the case of two instances of wheel flats).

Diameter	Wheel Flat Size (mm)		90° Interval	180° Interval
800	50	50	O	O
		62.5	O	O
		75	O	O
	62.5	50	O	X
		62.5	O	O
		75	O	O
	75	50	O	X
		62.5	O	X
		75	O	O
830	50	50	O	O
		62.5	O	O
		75	O	O
	62.5	50	O	X
		62.5	O	O
		75	O	O
	75	50	O	X
		62.5	O	X
		75	O	O
860	50	50	O	O
		62.5	O	O
		75	O	O
	62.5	50	O	X
		62.5	O	O
		75	O	O
	75	50	O	O
		62.5	O	O
		75	O	O

**Table 3:** Parameter values of STFT.

Parameter	Value
Window length	64
Window function	Hanning
Overlap	80%

**Table 4:** Dataset is of training data, validation data, and test data.

Data Type	EA	Ratio (%)
Train data	2988	60
Validation data	837	17
Test data	1152	23

**Table 5:** Learning results of the modified LeNet-5, ResNet-20, and modified MobileNet-V3.

Data Type	Accuracy (%)	AUC	Recall
Modified LeNet-5	97	0.9688	0.982
ResNet-20	89	0.8801	0.817
Modified MobileNet-V3	96	0.9573	0.915

**Table 6:** Learning results of the supervised and semi-supervised learning method.

Data Type	Accuracy (%)	AUC	Recall
Modified LeNet-5	97	0.9688	0.982
ResNet-20	89	0.8801	0.817
Modified MobileNet-V3	96	0.9573	0.915
Deep One Class Classification	94	0.9345	0.869

## Data Availability

The data in our paper is primarily obtained through direct experimentation. As these are results from our own experiments, the data cannot be disclosed to others. Additionally, any data not directly obtained through figures or tables has already been appropriately cited in the references.
